# A Qualitative Transcriptional Signature for Predicting CpG Island Methylator Phenotype Status of the Right-Sided Colon Cancer

**DOI:** 10.3389/fgene.2020.00971

**Published:** 2020-10-29

**Authors:** Tianyi You, Kai Song, Wenbing Guo, Yelin Fu, Kai Wang, Hailong Zheng, Jing Yang, Liangliang Jin, Lishuang Qi, Zheng Guo, Wenyuan Zhao

**Affiliations:** ^1^Department of Systems Biology, College of Bioinformatics Science and Technology, Harbin Medical University, Harbin, China; ^2^Department of Bioinformatics, Key Laboratory of Ministry of Education for Gastrointestinal Cancer, School of Basic Medical Sciences, Fujian Medical University, Fuzhou, China; ^3^Fujian Provincial Key Laboratory on Hematology, Fujian Medical University, Fuzhou, China

**Keywords:** right-sided colon cancer, CpG island methylator phenotype, the qualitative transcriptional signature, relative expression ordering, gene pairs

## Abstract

A part of colorectal cancer which is characterized by simultaneous numerous hypermethylation CpG islands sites is defined as CpG island methylator phenotype (CIMP) status. Stage II and III CIMP−positive (CIMP+) right-sided colon cancer (RCC) patients have a better prognosis than CIMP−negative (CIMP−) RCC treated with surgery alone. However, there is no gold standard available in defining CIMP status. In this work, we selected the gene pairs whose relative expression orderings (REOs) were associated with the CIMP status, to develop a qualitative transcriptional signature to individually predict CIMP status for stage II and III RCC. Based on the REOs of gene pairs, a signature composed of 19 gene pairs was developed to predict the CIMP status of RCC through a feature selection process. A sample is predicted as CIMP+ when the gene expression orderings of at least 12 gene pairs vote for CIMP+; otherwise the CIMP−. The difference of prognosis between the predicted CIMP+ and CIMP− groups was more significantly different than the original CIMP status groups. There were more differential methylation and expression characteristics between the two predicted groups. The hierarchical clustering analysis showed that the signature could perform better for predicting CIMP status of RCC than current methods. In conclusion, the qualitative transcriptional signature for classifying CIMP status at the individualized level can predict outcome and guide therapy for RCC patients.

## Introduction

Colorectal cancer (CRC) is the third most commonly diagnosed malignancy and the second leading cause of mortality in the world (Bray et al., 2018). The CpG island methylator phenotype-positive (CIMP+) tumor, which is characterized by vast hypermethylation of promoter CpG island sites, accounts for 17–20% of CRC (Jass, 2005; Kudryavtseva et al., 2016). Several studies indicated that the stage II and III CRC patients with CIMP+ status are associated with a better prognosis than CIMP−negative (CIMP−) CRC patients, and CIMP+ CRC patients cannot benefit from 5-Fluorouracil (5-FU)-based adjuvant chemotherapy (ACT; Ogino et al., 2009; Jover et al., 2011).

Currently, the CIMP status is commonly detected by methylation-specific polymerase chain reaction (PCR) and methylight techniques. The methylation-specific PCR detects five biomarkers with MINT1, MINT2, MINT31, CDKN2A (p16), and MLH1 (Issa, 2004), and the methylight detects five biomarkers with CACNA1G, IGF2, NEUROG1, RUNX3, and SOCS1 (Weisenberger et al., 2006). For each panel of CIMP markers, CRC is classified as CIMP+ if three or more CIMP markers are methylated which are also called as CIMP−high (CIMP−H). Besides, the others are classified as CIMP− which are also divided into CIMP−low (CIMP−L) if one or two CIMP markers are methylated and CIMP−0 if no methylated marker is observed (Jover et al., 2011; Min et al., 2011). Because CIMP−L patients have the same prognosis as CIMP−0 patients, and CIMP−L or CIMP−0 patients can benefit from 5-FU-based ACT (Juo et al., 2014), it is reasonable to group CIMP−L and CIMP−0 as CIMP− in our study. It is worth noting that the technologies commonly used could cause false-positive and false-negative results. The false-positive results arise from the incomplete bisulfite conversion, false priming, and the too low annealing temperature or too many used cycles (Kristensen et al., 2008). The false-negative results are caused by the insufficient amount of input DNA, DNA degradation during bisulfite treatment, low stability of single-strand DNA, and strand-specific PCR amplification (Liu et al., 2016; Advani et al., 2018). Currently, there is no golden standard with respect to technologies and CIMP markers for the detection of altered DNA methylation used to define CIMP status (Jia et al., 2016; Bae et al., 2017; Advani et al., 2018). Therefore, it is worthwhile to develop a credible signature for predicting CIMP status.

Nowadays, because of the cost-effective of transcriptome analysis and the regulatory relationships between the DNA methylation and gene expression, several quantitative transcriptional signatures have been developed for predicting the CIMP status of CRC patients (Siegfried and Simon, 2010; Moarii et al., 2015; Xi et al., 2017). The quantitative signatures are sensitive to the systematic inter-laboratory biases of microarray or RNA-sequencing experiments, especially batch effects, which are introduced by experimental conditions, regent dosages, microarray technology, and operational procedures (Leek et al., 2010; Qi et al., 2016), resulting in the failures in independent inter-laboratory data. In addition, the quantitative signatures would also be greatly affected by varied proportions of tumor epithelial cell in tumor tissues sampled from different tumor locations of the same patient (Cheng et al., 2017), partial RNA degradation during specimen storage and preparation (Chen et al., 2017), and amplification bias for minimum specimens even with about 15–25 cancer cells (Liu et al., 2017), which are common factors that can lead to failures in clinical applications. In contrast, the qualitative signatures based on relative expression orderings (REOs) of gene pairs within a sample are robust against the batch effects, different tumor locations, partial RNA degradation, and amplification bias (Zhao et al., 2016; Song et al., 2019), which could be directly applied to the sample at the individual level in clinical applications (Qi et al., 2016; Chen et al., 2017; Cheng et al., 2017; Liu et al., 2017; Li et al., 2019).

Consistent with the differences in anatomy location, the left-sided colon cancer (LCC) and right-sided colon cancer (RCC) have different embryonic developmental sites, genomic patterns and different clinical symptoms (Loupakis et al., 2015; Shen et al., 2015; Barton, 2017). Additionally, among the CIMP+ CRC, RCC has a significantly higher prevalence (87%) than LCC (13%) (Yamauchi et al., 2012). Thus, in this study, we developed a qualitative transcription signature for predicting CIMP status of stage II and III RCC at the individual levels. The performance of the signature was evaluated in four independent datasets by receiver operating characteristic (ROC) analysis. Meanwhile, based on the patients’ relapse-free survival (RFS), we provided evidence that the signature could perform better for identifying CIMP status of RCC than current methods.

## Materials and Methods

### Data and Preprocessing

The gene expression and methylation datasets for colon cancer used in this study were downloaded from the Gene Expression Omnibus database (GEO)^1^ and the ArrayExpress database,^2^ as described in detail in Tables 1, 2.

**TABLE 1:** The datasets detected CpG island methylator phenotype (CIMP) status in this study.

	GSE39582 (*n* = 510)	GSE39084 (*n* = 19)	GSE25070 (*n* = 22)	E-TABM-328 (*n* = 47)
**Stage**				
I	37	–	–	–
II	247	20	–	–
III	167	14	–	–
IV	59	–	–	–
**CIMP status**				
CIMP+	93	6	6	11
CIMP−	417	13	16	36
**Location**				
Right	210	19	13	22
Left	300	–	9	25

**CIMP detection**				

	**Methylight**	**Methylight**	**Methylight**	**Methylation-specific PCR**

**Adjuvant chemotherapy**				
Yes	296	–	–	–
No	201	–	–	–
NA	16	–	–	–
**Platform**	Affymetrix Human Genome U133 Plus 2.0 Array	Affymetrix Human Genome U133 Plus 2.0 Array	Illumina Human Ref-8v3.0 expression beadchip	Whole Human Genome Microarray 4x44K

**TABLE 2:** The datasets detected both gene expression and DNA methylation profiles in this study.

	GSE25070 (*n* = 22)	GSE25062 (*n* = 22)	GSE79793 (*n* = 26)	GSE79740 (*n* = 26)
				
Data type	Expression profiling	Methylation profiling	Expression profiling	Methylation profiling
**CIMP status**				
CIMP+	6	6	–	–
CIMP−	16	16		
**Platform**	Illumina Human Ref-8 v3. 0 expression beadchip	Illumina Human Methylation27 BeadChip	Illumina Human HT-12 WG-DASL V4. 0 R2 expression beadchip	Illumina Human Methylation450 BeadChip

The training dataset for extracting a REOs-based signature was GSE39582, including 64 CIMP+ and 117 CIMP− stage II and III RCC samples, which recorded the information of RFS of patients for further survival analyses. Because of the small sample size of RCC in GSE39084, GSE25070 and E-TABM-328, so the three cohorts including a total of 54 RCC samples were combined as the validation cohort to test the predictive signatures. Besides, we used the samples which detected both gene expression profiles and DNA methylation profiles (match GSE25070 to GSE25062 and match GSE79793 to GSE79794) to select the differentially methylated CpG sites between the CIMP+ and CIMP− samples predicted by the signature.

For data measured by the Affymetrix platform, we downloaded the raw mRNA expression data (CEL files) and used the Robust Multi-Array Average algorithm (Irizarry et al., 2003) for background adjustment. For data measured by the Illumina and Agilent platform, we directly downloaded the processed data (series matrix files). For each gene expression database, the rule of processing all probes was following: the expression measurements of multiple probes mapping to the same Entrez Gene ID were averaged to obtain a single measurement, and the probes that did not map to any Entrez Gene ID or mapped to multiple Entrez Gene IDs were discarded. For the gene methylation datasets, we only analyzed the 25014 CpG sites detected by both the 27K array and 450K array which were not targeted the X and Y chromosomes. Using methylated signal intensity (M) and unmethylated signal intensity (U), the DNA methylation level of each probe was calculated by *M*/(*U* + *M* + 100) (Dedeurwaerder et al., 2011).

### Differentially Methylated CpG Sites and Expressed Genes Analysis

For microarray data, we selected differential methylated CpG sites or differentially expressed (DE) genes between two classes of samples using the limma algorithm (Ritchie et al., 2015). The *P* values were adjusted by the Benjamini–Hochberg procedure for multiple testing to control the false discovery rate (FDR; Hochberg and Benjamini, 1990).

### Signature Development for Predicting CIMP Status of RCC

Firstly, for a gene pair, *i* and *j*, with expression values of *E*_*i*_ and *E*_*j*_, we used Fisher’s exact test (Crans and Shuster, 2008) to evaluate whether the frequency of a specific REO pattern (*E_*i*_ > E_*j*_* or *E_*i*_ < E_*j*_*) was significantly higher in the CIMP+ samples than the frequency in the CIMP− samples. The gene pairs which were detected with FDR < 0.01 were defined as CIMP−related gene pairs.

Secondly, because some genes appeared in multiple CIMP−related gene pairs, we narrowed down the number of gene pairs via a redundancy removal method. For a gene that appeared in multiple gene pairs, we only kept the gene pair with the largest frequency difference (FD) value and discarded others. The FD was calculated for each gene pair by the following formula.

*p*_*ij*_(*c*) = *P*(*E*_*i*_ > *E*_*j*_|*c*), c = 1, 2, the probabilities of observing *E_*i*_> E_*j*_* in each group.

*FD*_*ij*_ = *p*_*ij*_(1)−*p*_*ij*_(2), the FD value of a gene pair (*i*, *j*).

The bigger the FD value was, the more stable the difference of REOs between two groups of samples was. After that, we obtained a panel of gene pairs with no less than an FD cutoff with 0.01 spacing distance from the maximum to minimum. Finally, we selected the optimal vote rule for each gene panel according to their harmonic mean value (F-score) of sensitivity and specificity in predicted CIMP+ and CIMP− groups. A sample was labeled as CIMP+ if the REOs of at least k gene pairs in the panel of gene pairs were consistent with the specific patterns (*E_*i*_ > E_*j*_*) of the training samples, and vice versa. For each k ranging from 1 to the number of gene pairs in the panel of gene pairs, we could compute the corresponding F-score. The F-score was calculated by the following formula.

F-score=2×sensitivity×specificity÷(sensitivity+specificity)

We selected the k which could reach the largest F-score as the optimal vote rule for each panel of gene pairs. Finally, we selected the panel of gene pairs which reached the largest F-score as the signature.

### Sample Clustering

The Limma algorithm was performed to identify DE genes between the samples with predicted CIMP+ and CIMP− by the signature confirmed with the original CIMP status. Complete linkage hierarchical clustering analysis was performed to stratify RCC samples into two subgroups. The similarity of samples was evaluated by the Euclidean distance based on the expression measurements of DE genes.

### Statistical Analysis

The RFS is the period from the date of initial surgical resection until the date of the first occurrence of a new tumor event or the final documented data (censored). The Kaplan-Meier method and the log-rank test were used to evaluate the survival curve and compare the difference of survival curves, respectively (Bland and Altman, 2004). Univariable Cox proportional hazards regression model calculated the Hazard Ratio (HR) and the 95% confidence interval (95% CI; Harrell et al., 1996). The predictive performance of the signature was calculated by using the area under the curve (AUC) of the ROC curve analysis (McClish, 1989). The functional categories for enrichment analysis were downloaded from KEGG (Kanehisa et al., 2012). The hypergeometric distribution model was used to test whether a set of genes observed in a functional term was significantly more than what was expected by random chance. All statistical analyses were performed using the R 3.5.2 software package.^3^

## Results

### Identification of the Predictive Signature for CIMP Status of RCC

**FIGURE 1:**
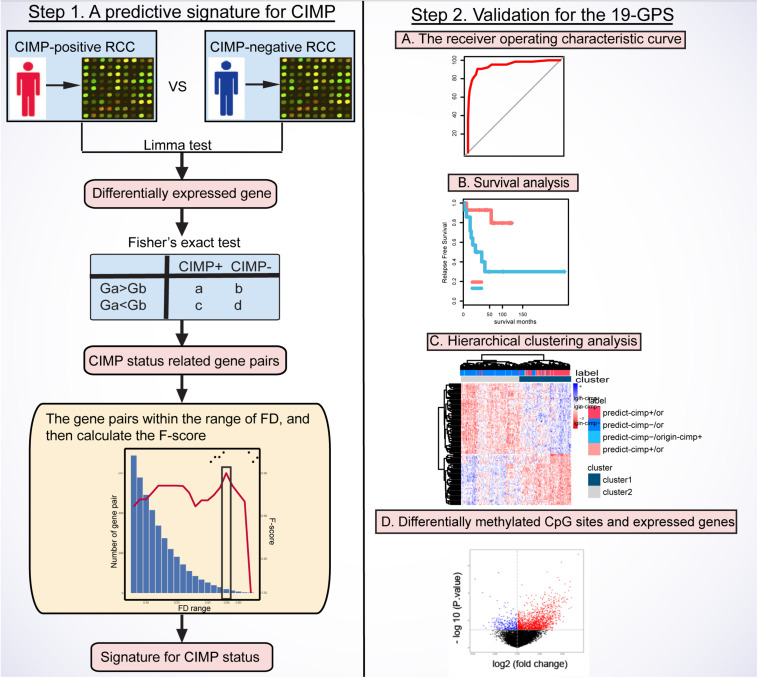
Flowchart of this study. CIMP, CpG island methylator phenotype; RCC, right-sided colon cancer; FD, frequency difference; F-score, harmonic mean value; 19-GPS, 19 gene pairs signatures.

**FIGURE 2:**
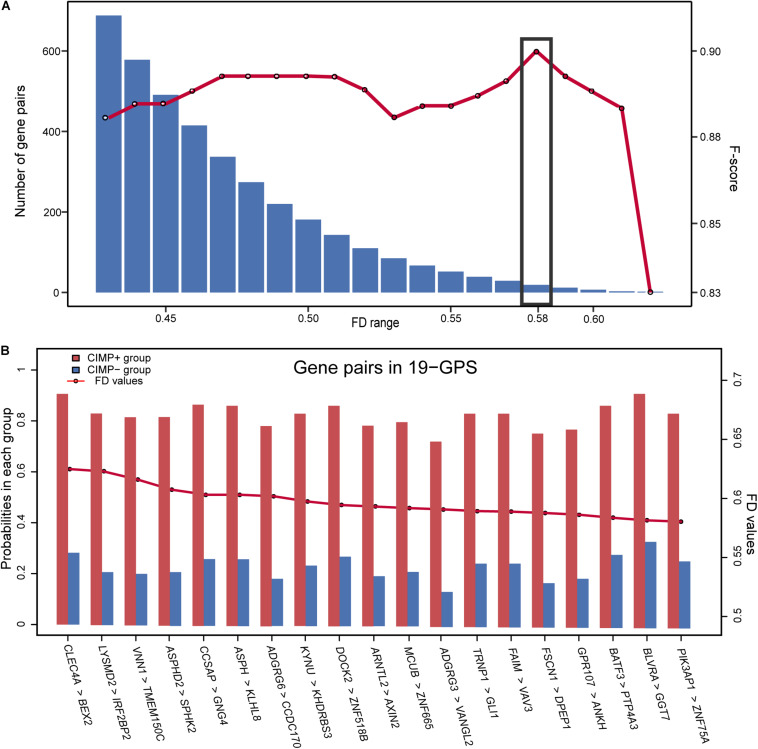
The F-score and number of the gene pairs within different range of FD values **(A)** and composition of 19-GPS **(B)**. The *x*-axis represents the range of FD value and the relative expression orderings (REOs) (gene1 > gene2) of 19-GPS, respectively.

Figure 1 describes the flowchart of this study. The GSE39582 dataset including the largest sample size of stage II and III RCC with CIMP status was used as the training data for selecting an REOs-based signature. Firstly, we identified 2209 DE genes between the 64 CIMP+ RCC samples and the 117 CIMP− RCC samples (limma test, FDR < 0.01). From all gene pairs consisting of at least one DE gene, we extracted 383,591 CIMP−related gene pairs whose specific REOs patterns occurred more frequently in the CIMP+ than in the CIMP− samples (Fisher’s exact test, FDR < 0.01). Then, 53 panels of gene pairs were found within different ranges of the FD value. After a redundancy removal process for each panel of gene pairs, we calculated the largest F-score with the optimal vote rule (Figure 2A, see section “Materials and Methods”). Finally, the 19 gene pairs, which obtained the largest F-score within the range of FD more than 0.58, were denoted as 19 gene pairs signatures (19-GPS) for predicting CIMP status of stage II and III RCC (Figure 2B).

**FIGURE 3:**
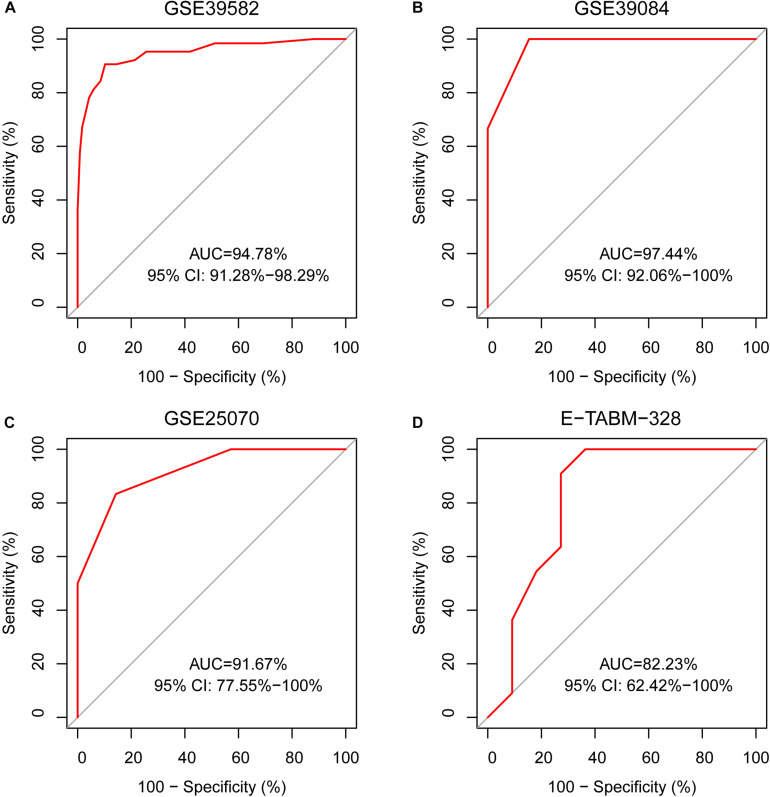
The ROC curves for 19-GPS in four independent datasets. **(A)** The right-sided colon cancer (RCC) of the training dataset, **(B)** The RCC of GSE39084, **(C)** The RCC of GSE25070, and **(D)** The RCC of E-TABM-328.

A sample was predicted as CIMP+ if the REOs of at least 12 gene pairs in 19-GPS voted for CIMP+; otherwise the CIMP−. According to the classification rule, the F-score of the signature in the training data was 0.91 (Table 3) with a sensitivity of 0.91 and a specificity of 0.90, and the AUC of the ROC curve was 0.95 (95% CI: 92.08–97.83%) (Figure 3A).

**TABLE 3:** The performance of 19-GPS for right-sided colon cancer (RCC) samples in the training and validation datasets.

	pre-CIMP+ (CIMP+:CIMP−)	pre-CIMP− (CIMP+:CIMP−)	Sensitivity	Specificity	*F*-score
GSE39582	70 (58:12)	111 (6:105)	0.91	0.90	0.95
GSE39084	11 (6:5)	8 (0:8)	1	0.62	0.76
GSE25070	6 (5:1)	7 (1:6)	0.83	0.86	0.85
E-TABM-328	13 (10:3)	9 (8:1)	0.91	0.73	0.81
Total RCC	100 (79:21)	135 (15:120)	0.84	0.85	0.85

*The CIMP+ and CIMP− represented the original CIMP status; pre-CIMP+ and pre-CIMP− represented the CIMP status predicted by 19-GPS.*

**FIGURE 4:**
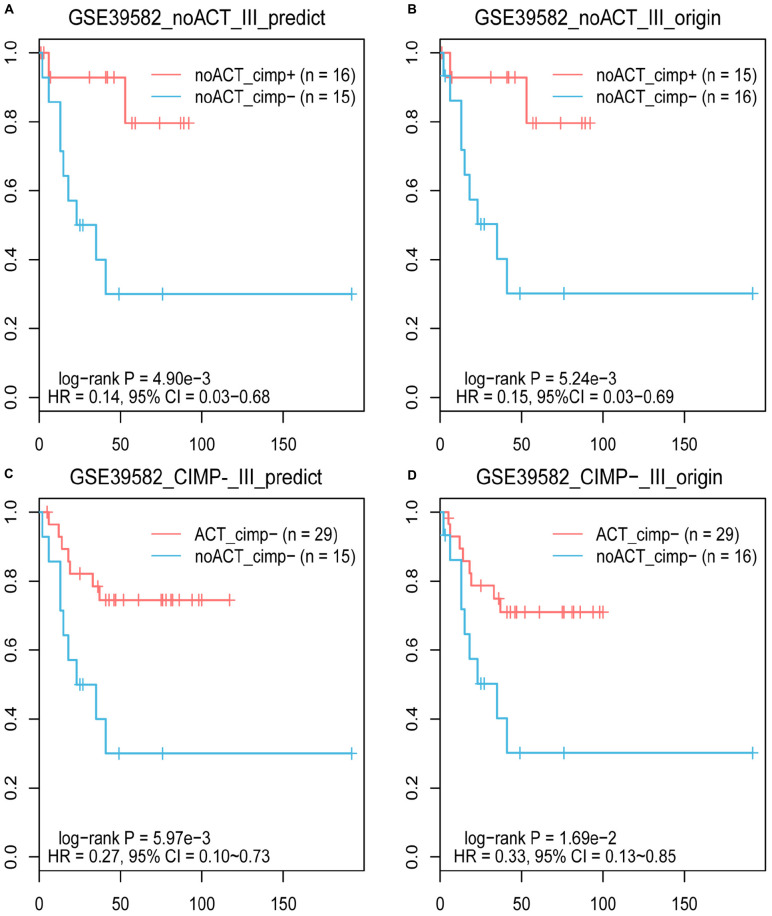
The Kaplan–Meier curves for the prediction of 19-GPS and original CIMP status in training dataset. **(A,B)** Stage III RCC of the CIMP+ and CIMP− patients treated with surgery alone. The red and blue lines represent the CIMP+ and CIMP− patients treated with surgery alone, respectively. **(C,D)** All of stage III RCC of the CIMP− patients. The red and blue lines represent the CIMP− patients receiving 5-FU-based ACT and treated with surgery alone, respectively.

Based on the knowledge that stage II and III CIMP+ RCC patients treated with surgery alone have better prognoses than CIMP− RCC patients (Ogino et al., 2009; Jover et al., 2011), we evaluated the reliability of 19-GPS through survival analysis. In the training dataset containing 31 samples of stage III RCC patients treated with surgery alone, one of the 16 original CIMP− samples was reclassified as CIMP+ by 19-GPS (Supplementary Table 1). The survival analysis showed that the RFS of the 16 predicted CIMP+ patients was significantly longer than the 15 predicted CIMP− patients (log-rank *P* = 4.90e-3, HR = 0.14, 95% CI = 0.03–0.68, Figure 4A), which was more significant than the difference between patients with the original CIMP status due to the reclassified sample (log-rank *P* = 5.24e-3, HR = 0.15, 95% CI = 0.03–0.69, Figure 4B). It is also known that stage III CIMP− RCC patients treated with 5-Fu-based ACT have better outcomes than patients treated with surgery alone (Jover et al., 2011). In the 41 stage III RCC samples of training data for patients receiving 5-Fu-based ACT, 2 of the 29 original CIMP− samples were reclassified as CIMP+ by 19-GPS, and 2 of the 12 original CIMP+ samples were reclassified as CIMP− (Supplementary Table 1). The survival analysis showed that the RFS of the 29 predicted CIMP− patients receiving 5-Fu-based ACT was significantly longer than the 15 predicted CIMP− patients treated with surgery alone (log-rank *P* = 5.97e-3, HR = 0.27, 95% CI = 0.10–0.73, Figure 4C), which was more significant than the different between original CIMP− patients treated with 5-FU-based ACT and surgery alone (log-rank *P* = 1.69e-2, HR = 0.33, 95% CI = 0.13–0.85, Figure 4D). The survival analysis validated that 19-GPS could perform better for predicting CIMP status of stage II and III RCC patients than current methods.

**FIGURE 5:**
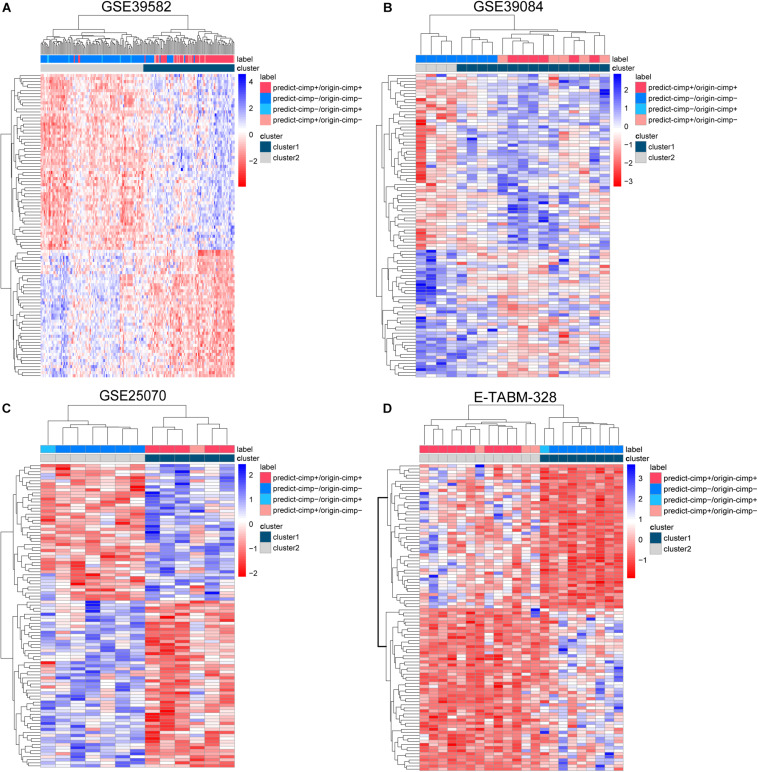
The complete linkage hierarchical clustering analysis of the RCC samples in four independent datasets. **(A)** GSE39582, **(B)** GSE39084, **(C)** GSE25070, and **(D)** E-TABM-328 based on the differentially expressed genes between the signature-confirmed CIMP+ and CIMP− samples. predict-CIMP/origin-CIMP, predict-CIMP represented the predicted CIMP status by 19-GPS and origin-CIMP represented the original CIMP status.

There were 12 CIMP− and 6 CIMP+ samples reclassified by 19-GPS in the total of stage II and III RCC of training dataset. We contrasted the gene expression patterns of the 18 signature-disconfirmed samples with the 163 signature-confirmed samples through hierarchical clustering analysis. Firstly, we identified 4685 DE genes between the 58 signature-confirmed CIMP+ samples and the 105 signature-confirmed CIMP− samples in the training dataset (limma test, FDR < 0.01). Secondly, using the expression measurements of the top 100 significant DE genes, the samples were classified into two subgroups using the complete linkage hierarchical clustering analysis based on the Euclidean distance (Figure 5A). The results showed that all of the samples reclassified as CIMP+ and CIMP− were clustered with the group of signature-confirmed CIMP+ and CIMP− samples, respectively. The gene expression patterns validated the correctness of 19-GPS in training dataset.

### Validation of 19-GPS in Independent Datasets

In three validation datasets (GSE39084, GSE25070 and E-TABM-328) of RCC samples, the CIMP status of samples was predicted based on 19-GPS. In GSE25070, *TMEM150C* and *CCDC170* included in 19-GPS were not detected by Illumina Human Ref-8v3.0 expression beadchip, which resulted in 17 gene pairs available for classification. Then we observed that the classifier of 17 gene pairs achieved the largest F-score when requiring that at least 10 of 17 gene pairs voted for CIMP+ determination in the training dataset, so the vote rule was regarded as the optimal vote rule in GSE25070. Similarly, in E-TABM-328, 18 gene pairs were detected by Whole Human Genome Microarray 4x44K, and CIMP+ determination could be voted by at least 11 of 18 gene pairs as the optimal vote rule. The F-score of the signature were 0.76, 0.85, and 0.81 in GSE39084, GSE25070, and E-TABM-328. The AUC of ROC were 97.44% (95% CI: 91.37–100%), 91.67% (95% CI: 61. 68–100%) and 82.23% (95% CI: 70.59–100%) (Figures 3B–D).

Because the therapeutic and survival information was unavailable in three validation datasets, we compared the gene expression patterns of the signature-disconfirmed samples with the signature-confirmed samples through hierarchical clustering analysis in the validation datasets. Using the expression levels of the top 100 significant DE genes between the signature-confirmed CIMP+ and CIMP− samples (limma test, FDR < 0.01), the samples were classified into two subgroups using the hierarchical clustering analysis (Figures 5B–D). In GSE39084, the result showed 4 of 5 CIMP− samples reclassified as CIMP+ by our signature were clustered with the group of signature-confirmed CIMP+ samples. The similar results were observed in GSE25070 and E-TABM-328 that all of the samples reclassified as CIMP+ and CIMP− were clustered with the group of signature-confirmed CIMP+ and CIMP− samples, respectively. These results provided transcriptional evidence of the correctness of the prediction of 19-GPS.

### The Differentially Methylated CpG Sites and Expressed Genes Between CIMP+ and CIMP− Samples

**FIGURE 6:**
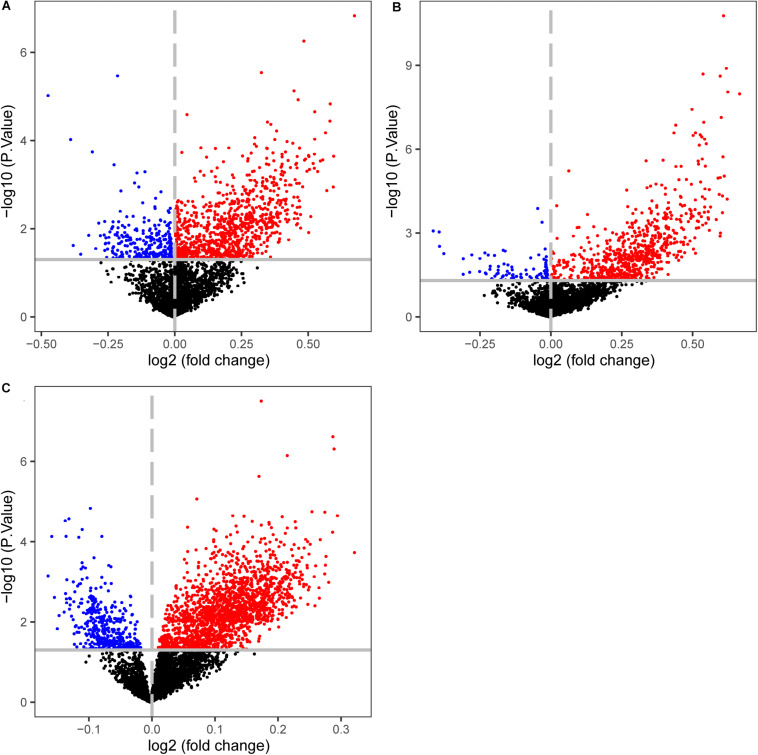
Volcano plots of the differentially methylated CpG sites between CIMP+ and CIMP− samples. **(A)** The samples with predicted CIMP status by 19-GPS in GSE25062. **(B)** The samples with original CIMP status in GSE25062. **(C)** The samples with predicted CIMP status by 19-GPS in GSE79740. The log2 (fold change) beta value difference in DNA methylation between the samples with CIMP+ and CIMP− status is plotted on the *x*-axis, and the *P* value (−1 × log10 *P* value) for limma test of differences between the two subtypes is plotted on the *y*-axis. The CpG sites which are significantly different and log2 (fold change) > 0 between the two subtypes are shown in red, and the CpG sites which are significantly different and log2 (fold change) < 0 are shown in blue.

The CIMP+ status is characterized by high frequency of promoter hypermethylation whose regions almost locate in tumor suppressor genes (Loupakis et al., 2015). We used the datasets detected both gene expression and DNA methylation profiles to select the differentially methylated CpG sites between predicted CIMP+ and CIMP− samples (match GSE25070 to GSE25062 and match GSE79793 to GSE79794). The CIMP status predicted by 19-GPS in GSE25070 was used in GSE25062. Then, the 1581 hypermethylated CpG sites were selected between the predicted CIMP+ and CIMP− samples in GSE25062 (limma test, *P* < 0.05, Figure 6A). The hypermethylated CpG sites located in the regions of 26 tumor suppressor genes which were downloaded from The Cancer Gene Census containing 316 tumor suppressor genes.^4^ Meanwhile, the 1147 hypermethylated CpG sites were selected between original CIMP status samples in GSE25062, and they were located in the regions of 15 tumor suppressor genes (limma test, *P* < 0.05, Figure 6B). The results showed that the predicted CIMP+ samples had much more hypermethylated CpG sites and tumor suppressor genes than the original CIMP+ samples.

Then, we calculated the number of hypermethylated CpG sites and tumor suppressor genes of predicted CIMP+ samples based on the same method in GSE79793 and GSE79740. Compared with the predicted CIMP− samples, the predicted CIMP+ samples had 3124 hypermethylated CpG sites which were located in the regions of 57 tumor suppressor genes, (limma test, *P* < 0.05, Figure 6C). Because the samples had no original CIMP labels in above datasets, we could not assess the difference of the number of hypermethylated CpG sites and tumor suppressor genes between the predicted and original CIMP status. Moreover, the 552 hypermethylated CpG sites between predicted CIMP+ and CIMP− samples were identified in both GSE25062 and GSE79740, which did not randomly distribute among all of the hypermethylated CpG sites (*P* < 2.2e-16, Hypergeometric test).

Besides, we selected 4771 DE genes between the predicted CIMP+ and CIMP− samples, which were more than 2209 DE genes among the original samples in the training dataset (limma test, FDR < 0.05). This indicated that the differences in methylation and gene expression patterns between the predicted CIMP+ and CIMP− samples were more significant than the original samples. In conclusion, the differentially methylated CpG sites and expressed genes analysis provided the evidence that the characteristic of predicted CIMP status of samples conformed to the truly biological properties.

### The Robustness Against Varied Proportions of Tumor Epithelial Cell

Some reports show the qualitative signatures based on REOs of gene pairs are robust against the varied proportions of tumor epithelial cells (Cheng et al., 2017). To validate the robustness of 19-GPS, our laboratory collected 13 fresh-frozen primary tumor tissue samples through surgical excision. Fresh-frozen primary tumor tissue samples were retrospectively collected at Union Hospital of Fujian Medical University. And the 13 solid tumor tissue samples were from five patients whose excisions were from different sampling positions with different information of “percentage of tumor cells” as shown in Table 4. The institutional ethical review boards of Union Hospital of Fujian Medical University approved the protocol, and all patients signed informed consents before sample collection. And we used the fragments per kilobase of exon model per million mapped fragments to quantify the gene expression level from RNA sequencing data. Then, we used 16 gene pairs available for 19-GPS to predict the CIMP status of 13 samples. And the gene expression levels of 19-GPS were detailed in (Supplementary Table 2). There were 4 of 5 patients containing samples with different percentage of tumor cells predicted the same CIMP status, and 2 of 3 samples of the one remaining patient were also predicted the same CIMP status (Table 4). Because the different tumor tissue samples, which were from the same patient whose excisions were from different sampling positions with different information of “percentage of tumor cells,” were predicted the same CIMP status by 19-GPS. Therefore, the result confirmed that CIMP status predicted by 19-GPS was not affected by the different percentage of tumor cells of samples.

**TABLE 4:** The predicted CIMP status of samples with different percentage of tumor cells.

Sample ID	Percentage of tumor cells (%)	Predicted CIMP status
HCF1	40	Negative
HCF2	100	Negative
HCF3	100	Negative
LGL1	50	Negative
LGL2	90	Positive
LGL3	90	Positive
SDL1	100	Negative
SDL2	100	Negative
WCY1	60	Negative
WCY2	100	Negative
WCY3	100	Negative
ZCH1	70	Negative
ZCH3	40	Negative

## Discussion

In this study, we developed a robust qualitative transcriptional signature consisting of 19-GPS to individually identify the CIMP status for stage II and III RCC. We also tried to develop a signature to predict CIMP status for stage II and III LCC. However, the prevalence rate of CIMP+ among LCC was only 2.04–6.67% in the training and validation datasets (Supplementary Table 3), and the statistics showed that the prevalence rate is about 2.67% in several studies (Natsume et al., 2018). There were so few LCC CIMP+ samples that we could not train or validate a signature to predict the CIMP status for LCC samples. During the process of developing the gene pairs signature, the aim of selecting DEGs was to reduce the number of gene pairs by the local optimization method. However, the development of gene pairs signature was influenced by the methods and cutoff for selecting DEGs. If all of the genes in gene expression profile were combined with each other, this global optimization method would lead to the overfitting result and the time of calculation process would be huge. After considering the feature of two methods, we decided to extract DEGs during the developing signature.

Some researches indicated several genes consisting of gene pairs had important roles during the process of tumor initiation and development. For example, among the CIMP+ samples, the gene expression of FSCN1 was higher than DPEP1 in the gene pair of FSCN1 > DPEP1. Some articles confirmed over-expression of FSCN1 in a variety of tumors usually correlates with high-grade, extensive invasion, distant metastasis, and poor prognosis (Chiyomaru et al., 2010). Meanwhile, loss of expression of DPEP1 as a tumor suppressor gene is associated with colorectal cancer and Wilms’ tumor (Green et al., 2009). Moreover, after identifying DE genes in training dataset, the functional enrichment analysis showed that the 4771 DE genes between the predicted CIMP+ and CIMP− samples were significantly enriched in 55 KEGG pathways (see section “Materials and Methods”) (FDR < 0.05, hypergeometric distribution, Supplementary Table 4). Especially, some cancer-associated pathways for metabolic pathway (La Vecchia and Sebastian, 2019), cell cycle pathway (Tominaga et al., 1997), and apoptosis pathway (Stoian et al., 2014) were significantly enriched. Among the 55 significantly enriched pathways, the mismatch repair pathway plays a critical role in maintaining the integrity and stability of the genome (Liu et al., 2019). And the p53 signaling pathway can regulate angiogenesis and metastasis, which is closely related to the progression and outcome of CRC (Slattery et al., 2019).

The association of CIMP status and the outcome was similar among stage II and III patients, but only stage III patients had a significant difference of survival analysis in the training dataset (Ogino et al., 2009). This may be due to the fact that the stage II patients had too much censored data to analyze in the training dataset. It is well known that the molecular marker consisting of CIMP and microsatellite instability (MSI) status can more accurately predict the outcome of CRC patients treated with surgery alone, compared with the molecular marker consisted of CIMP or MSI status alone (Ogino et al., 2009; Shiovitz et al., 2014). In the training dataset, we divided stage III RCC patients treated with surgery alone into four groups: CIMP+ with MSI-high (MSI-H) group, CIMP+ with microsatellite stability (MSS) group, CIMP− with MSI-H group and CIMP− with MSS group. We observed that the RFS of predicted CIMP+ with MSI-H group of patients treated with surgery alone was significantly longer than the others (log-rank *P* = 2.39e-2, Supplementary Figure 1A). After dividing samples into four categories, although the sample size was small in four groups, the survival difference between the predicted CIMP patients was more significant than original CIMP patients due to the one reclassified sample (log-rank *P* = 2.50e-2, Supplementary Figure 1B).

Some studies found that several genes consisted of 19-GPS were hypermethylated status, which played important roles during the process of tumor development. For example, as the component of 19-GPS, the expression of CLEC4A is higher than BEX2 among the CIMP+ samples. Some researchers found that BEX2 was silenced in all tumor specimens and exhibited extensive promoter hypermethylation, and viral-mediated re-expression of BEX2 led to increased sensitivity to chemotherapy-induced apoptosis and potent tumor suppressor effects in vitro and in a xenograft mouse model (Foltz et al., 2006).

Our laboratory proposes the concept of “a sequence for all,” which is composed by a series of qualitative transcriptional signatures for the prognostic and predictive biomarkers of CRC, including identifying micro-metastasis after surgery, 5-FU-based ACT benefit of high relapse risk patients, MSI status for CRC patients and so on (Zhao et al., 2016; Song et al., 2019). The qualitative transcriptional signature for predicting CIMP status in this study could combine with the other panels to predict the prognosis and guide the optimal therapy for CRC patients in clinical application.

## Conclusion

In summary, the qualitative transcriptional signature could robustly predict the CIMP status of stages II and III RCC at the individualized levels. The CIMP status predicted by 19-GPS can evaluate the outcome and guide the therapy for stage II and III RCC patients treated with surgery alone. The robustness and simplicity of the REO-based signature would make it convenient in clinical settings and worthy to further validate in a prospective clinical trial.

## Data Availability Statement

All training and validation datasets analyzed in this study were downloaded from the public database: GEO and Arrayexpress. The data analyzed during the analysis of robustness against varied proportions of tumor epithelial cell are included in Supplementary Table 2.

## Ethics Statement

The studies involving human participants were reviewed and approved by The Institutional Ethical Review Boards of Union Hospital of Fujian Medical University. The patients/participants provided their written informed consent to participate in this study.

## Author Contributions

WZ and ZG conceived the idea. TY conceived and designed the experiments and wrote the manuscript, KS and LQ designed the experiments. WG and YF analyzed the data. KW and HZ performed the experiments. JY and LJ helped in writing the manuscript. All authors approved the final version.

## Conflict of Interest

The authors declare that the research was conducted in the absence of any commercial or financial relationships that could be construed as a potential conflict of interest.

## Footnotes

1 http://www.ncbi.nlm.nih.gov/geo/

2 https://www.ebi.ac.uk/arrayexpress/

3 http://www.r-project.org/

4 https://cancer.sanger.ac.uk/census

## Supplementary Material

Supplementary Figure 1 The Kaplan-Meier curves of RFS of the CIMP with MSI groups identified by 19-GPS and original labels in training database. (A,B) All of stage III RCC of CIMP+ with MSI-H group, CIMP+ with MSS group, CIMP− with MSI-H group and CIMP− with MSS group treated with surgery alone.

Supplementary Table 1 The classification of 19-GPS in stage III RCC of GSE39582 in detail.

Supplementary Table 2 The gene expression values of 19-GPS in CRC samples with different percentages of tumor cells.

Supplementary Table 3 The number of CIMP+ and CIMP− LCC in the training and validation datasets.

Supplementary Table 4 Enrichment analysis for DE genes.

## Abbreviations

CIMP: CpG island methylator phenotype
CIMP+: CIMP − positive
CIMP−: CIMP − negative
CRC: colorectal cancer
RCC: right-sided colon cancer
LCC: left-sided colon cancer
REOs: relative expression orderings
19-GPS: 19 gene pairs signatures
F-score: harmonic mean value
FD: frequency difference
5-FU: 5-Fluorouracil
ACT: adjuvant chemotherapy
PCR: methylation-specific polymerase chain reaction
CIMP − H: CIMP − high
CIMP − L: CIMP − low
ROC: receiver operating characteristic
AUC: area under the curve
RFS: relapse-free survival
HR: hazard ratio
MSI: microsatellite instability
MSI-H: MSI-high
MSS: microsatellite stability
GEO: Gene Expression Omnibus
DE: differentially expressed
FDR: false discovery rate.
