# Supplementary figures and images for: A Qualitative Transcriptional Signature for Predicting CpG Island Methylator Phenotype Status of the Right-Sided Colon Cancer
Source: Front Genet. 2020 Oct 29;11:971. doi: 10.3389/fgene.2020.00971 (PMC7658404; doi:10.3389/fgene.2020.00971)

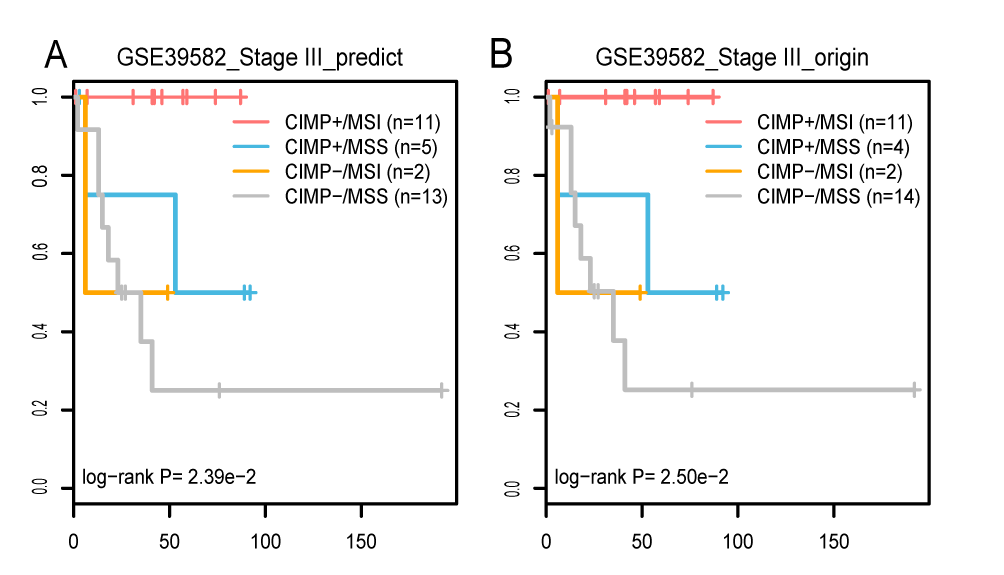

Supplement: Supplementary Figure 1 — The Kaplan-Meier curves of RFS of the CIMP with MSI groups identified by 19-GPS and original labels in training database. (A,B) All of stage III RCC of CIMP+ with MSI-H group, CIMP+ with MSS group, CIMP− with MSI-H group and CIMP− with MSS group treated with surgery alone. [file Image_1.TIF]
